# A systematic stepwise optimization framework for rapid multi-analyte UPLC–MS/MS plasma analysis with integrated sustainability assessment

**DOI:** 10.1038/s41598-026-63587-9

**Published:** 2026-07-28

**Authors:** Amira E. Abd-Elnabi, Amr M. Mahmoud, Dina A. El Mously, Omnia A. El-Naem

**Affiliations:** 1 Postgraduate Program in Pharmaceutical Analytical Chemistry, Faculty of Pharmacy, Cairo University, Cairo, 11562 Egypt; 2 Pharmaceutical Analytical Chemistry Department, Faculty of Pharmacy, October University for Modern Sciences and Arts (MSA), 6th October City, 11787 Egypt; 3 Pharmaceutical Analytical Chemistry Department, Faculty of Pharmacy, Cairo University, Kasr El-Aini Street, Cairo, 11562 Egypt

**Keywords:** UPLC–MS/MS, Stepwise optimization guidance model, Rifaximin, Ciprofloxacin, Fluconazole, Chemistry, Environmental sciences

## Abstract

A stepwise optimization guidance model for rapid multi-analyte UPLC–MS/MS is proposed to report recurring limitations in bioanalytical method development. The model involves six stages: (1) physicochemical profiling, (2) extraction strategy selection, (3) chromatographic optimization, (4) mobile phase selection, (5) method validation, and (6) greenness evaluation. The applicability of this framework was demonstrated by determining Rifaximin, Ciprofloxacin, and Fluconazole in spiked human plasma, using Ibuprofen as an internal standard. Physicochemical profiling guided the selection of ionization mode and prediction of solubility behavior. Based on the model recommendations, liquid–liquid extraction using dichloromethane was selected to achieve effective analyte recovery. Chromatographic optimization and mobile phase selection through the proposed framework supported the use of rapid isocratic separation on a C18 column using a methanol/water mixture (95:5, v/v) as mobile phase. The method validation, performed as an integral stage of the workflow according to regulatory guidelines, demonstrated exceptional linearity (r² ≥ 0.999). The final stage of the model involved evaluating environmental applicability using AGSA and EPPI analyses. The proposed sequential framework is intended to serve as a methodological reference for analysts employing LC–MS/MS platforms, to establish a structured and reproducible analytical workflow that advances ecological sustainability in multi-analyte LC–MS/MS bioanalytical applications.

**Supplementary Information:**

The online version contains supplementary material available at 10.1038/s41598-026-63587-9.

## Introduction

**Fig. 1:**
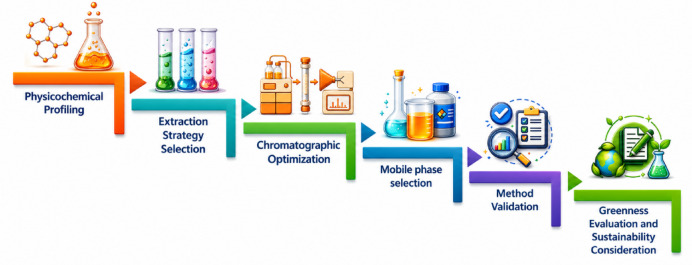
Stepwise optimization guidance model for rapid multi-analyte UPLC–MS/MS.

**Fig. 2:**
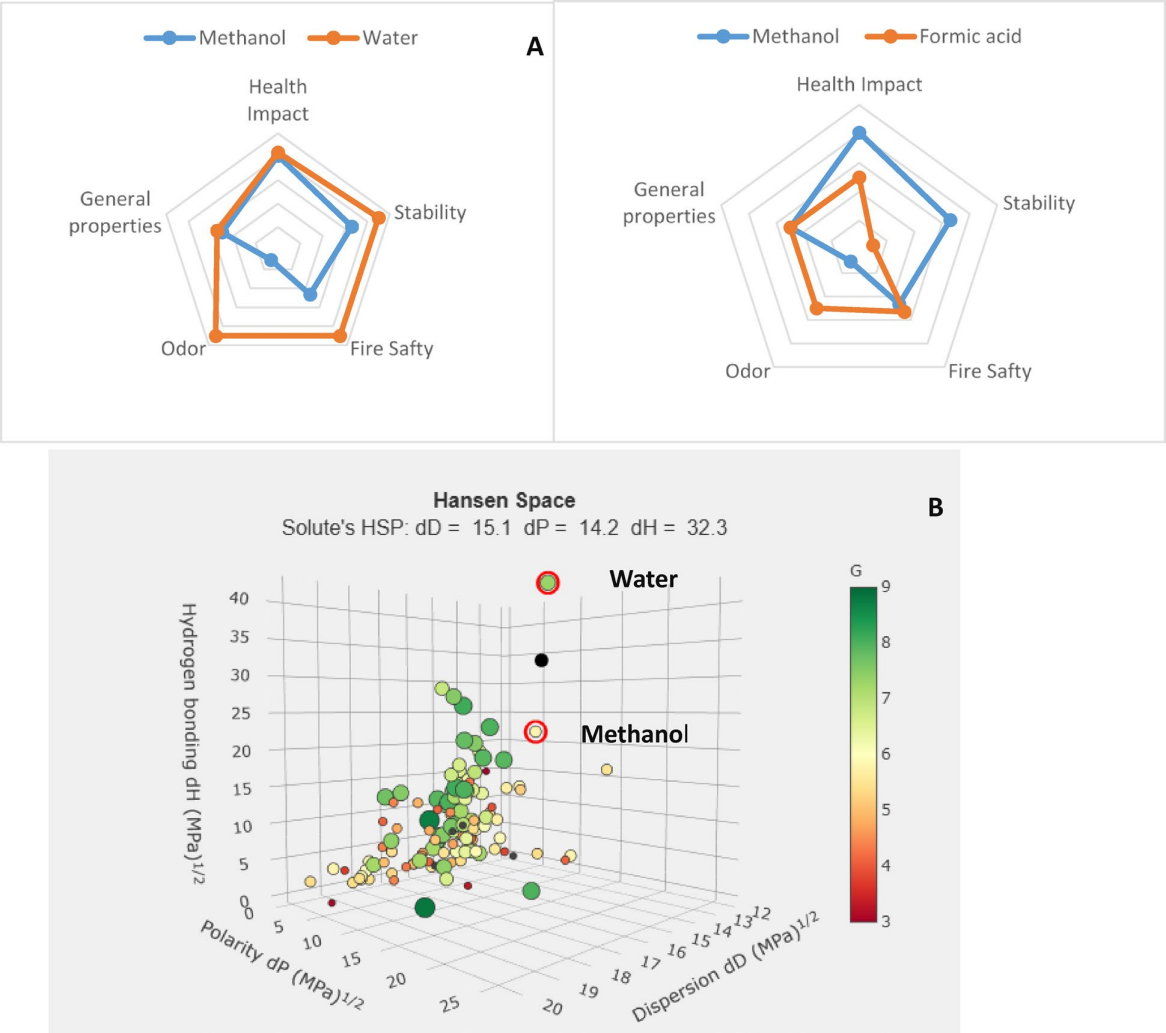
(**a**) Comparative greenness assessment and mobile phase selection based on spider diagram analysis. (**b**) Green solvent selection (GSST).

The combination of ultra-performance liquid chromatography and tandem mass spectrometry (UPLC–MS/MS) has advanced as a promising analytical technique for quantitative bioanalysis, valued for its particular sensitivity, selectivity, and rapid throughput^1^. Even with its extensive analytical capabilities, UPLC–MS/MS methods continue to meet repetitive technical challenges^2^. Most published UPLC–MS/MS studies focus on final validation results without thoroughly explaining how the identified limitations were addressed during method development^3^. The reasoning behind selections such as ionization polarity, chromatographic conditions, extraction technique, and MRM transitions is briefly mentioned^3^. Electrospray ionization-based systems is highly influenced by matrix effects and ion suppression, which are considered the most recurring obstacles in method regulation. Other issues may affect method robustness, such as crosstalk between multiple reaction monitoring (MRM) transitions, analyte degradation throughout sample preparation, and in-source fragmentation, especially in high-speed multi-analyte determinations that employ polarity switching^4^. Further major concerns for UPLC-MS/MS quantification include eco-sustainability and environmental assessment. Standard plasma UPLC–MS/MS extraction depend heavily on organic solvents like acetonitrile and formic acid, producing substantial chemical waste, particularly in high-throughput environments^5^. Although green analytical chemistry principles have been commonly discussed in the literature, sustainability considerations are rarely incorporated into bioanalytical LC–MS/MS method development^6^. Collectively, these limitations highlight the need for a structured optimization framework that reports recurring analytical challenges such as matrix effects, extraction technique selection, and eco-sustainability. A systematic workflow as described in (Fig. 1) that involves (1) physicochemical profiling, (2) extraction strategy selection, (3) chromatographic optimization, (4) mobile phase selection, (5) method validation, and (6) greenness assessment could meaningfully improve the reproducibility, sensitivity, and robustness of rapid multi-analyte UPLC–MS/MS plasma assays. The proposed stepwise optimization guidance model provides a structured framework for developing rapid multi-analyte UPLC–MS/MS methods through six interconnected stages. First, physicochemical profiling evaluates key analyte properties, including pKa, lipophilicity, solubility, and ionization behavior, to guide subsequent analytical decisions. Second, extraction strategy selection considers analyte–matrix interactions and compares sample-preparation approaches to maximize recovery while minimizing matrix effects. Third, chromatographic optimization focuses on stationary-phase selection and critical separation variables to achieve rapid and reliable multi-analyte resolution. Fourth, mobile-phase selection balances chromatographic performance, MS compatibility, and environmental considerations. Fifth, method validation confirms analytical reliability through relevant performance characteristics and recognized validation guidelines. Finally, greenness and sustainability evaluation provides a multidimensional assessment using RAPI, BAGI, MoGAPI, AGREE, AGSA, and RGB12, integrating analytical performance, practical applicability, environmental impact, and overall method whiteness. This study proposes and validates a structured optimization workflow to minimize common UPLC-MS/MS limitations in multi-analyte plasma analysis, using Rifaximin (RIF), Ciprofloxacin (CIP), and Fluconazole (FLU) as a representative chemically diverse panel. This panel represents a challenging physicochemical spectrum, making it suitable to demonstrate a structured optimization workflow. RIF, CIP, and FLU are widely used agents in the management of gastrointestinal infections and dysbiosis-related disorders. RIF is a semi-synthetic rifamycin derivative (Figure. S1) that acts through inhibition of bacterial RNA polymerase and is prescribed for traveler’s diarrhea, irritable bowel syndrome, small intestinal bacterial overgrowth, and hepatic encephalopathy^7^. CIP is a second-generation fluoroquinolone (Figure. S1) that inhibits DNA gyrase and topoisomerase IV and is effective against a broad spectrum of gram-positive and gram-negative pathogens^8^. FLU, a triazole antifungal (Fig. 2, S1), inhibits ergosterol synthesis via interaction with fungal 14-α-demethylase and is commonly used for systemic and mucosal fungal infections^9^. Combination of small intestinal fungal overgrowth (SIFO) and small intestinal bacterial overgrowth (SIBO) results in overlapping risk factors such as immunosuppression, chronic antibiotic use, and impaired gut motility. While RIF and FLU are well-established first-line therapies for SIBO and SIFO, respectively, in cases where both bacterial and fungal overgrowth are confirmed, a combination approach using rifaximin to target bacterial dysbiosis and fluconazole to address fungal proliferation may be clinically justified. This dual-targeted strategy offers a rational and potentially effective means of alleviating complex gastrointestinal symptoms when monotherapy is insufficient^10^. RIF and CIP are used for the treatment of gut dysbiosis, which is an alteration in the number or composition of gut micro-organisms: bacteria, fungi, viruses, protozoa, and archaea. This disruption can affect the body’s homeostasis, leading to inflammatory bowel syndrome (IBS)^11^. IBS has major symptoms that reduce personal quality of life, causing diarrhea, abdominal pain, bloating, gas that leads to malabsorption, anemia, and nutrient deficiency^12^. This major alteration in microbial gastrointestinal balance may lead to fungal overgrowth that increases IBS symptoms, which comes with the role of FLU to recover its balance and to manage antibiotic-induced dysbiosis. A combination of RIF, CIP, and FLU has been used for treating abdominal discomfort in cases of recurrent fungal and bacterial infections, as reported in this clinical case study^13^.

Stepwise Optimization Guidance Model for Rapid Multi-Analyte UPLC–MS/MS

Step 1: Physicochemical Profiling.

Step 2: Extraction Strategy Selection.

Step 3: Chromatographic Optimization.

Step 4: Mobile phase selection.

Step 5: Method Validation.

Step 6: Greenness Evaluation and Sustainability Consideration.

### **Step 1: physicochemical profiling**

Pre-development mapping involves systematic evaluation of analyte physicochemical properties and anticipated matrix interactions before experimental optimization begins. Parameters such as solubility, pKa, logP, and protein binding are used to predict extraction efficiency, ionization behavior, and susceptibility to matrix effect as Electrospray Ionization (ESI) requires the molecule to be charged^14^. If the mobile phase pH is close to the analyte’s pKa, the ionization will be unstable, leading to poor sensitivity and reproducibility. While logP, is used to measure the hydrophilicity or lipophilicity of the chosen antibiotic^15^. Early identification of potential risks such as ion suppression, co-elution with phospholipids, and sensitivity alterability supports rational choice of chromatographic strategies and extraction in addition to experimental adjustment^16^.

### Step 2: extraction strategy selection

Sample preparation is considered the primary barrier between complex biological matrices and the mass spectrometer. Selection of liquid–liquid extraction, protein precipitation, or solid-phase extraction should depend on predicted matrix complexity and required sensitivity^17^. If the analyte is highly polar, it will be challenging to extract from plasma using Liquid-Liquid Extraction (LLE), which applies non-polar organic solvents. Solid Phase Extraction (SPE) or simple precipitation will be more suitable^18^. LLE and SPE typically provide superior removal of endogenous phospholipids compared with protein precipitation, thereby reducing the risk of ion suppression^19^. Modification of sample pH to favor the neutral form of ionizable compounds enhances partitioning efficiency and recovery^20^. As for Partitioning efficiency, by adjusting the pH to make the drug neutral, 99% of it will be forced into the extraction solvent, leading to high recovery. Regulated evaluation of recovery and matrix factor across concentration levels is recommended to confirm extraction robustness^21^.

### Step 3: chromatographic optimization

Ultra-performance chromatographic methods are well known of low analysis time and solvent consumption, which increases the risk of co-elution with endogenous matrix components^22^. Simultaneous analysis of compounds involving different ionization polarities requires polarity switching during MS acquisition under isocratic conditions^23^. Retention and electrospray response are highly influenced by eluent composition and its pH, in addition to optimization of speed, resolution, and ionization stability^24^. Therefore, evaluation of number of MRM transitions, cycle time, and sufficient data points per peak are highly important to maintain quantitative reliability^25^.

### Step 4: mobile phase selection

The proposed optimization strategy is dedicated to highlight the impact of the mobile phase selection due to its central role in controlling both chromatographic behavior and mass spectrometric response while simultaneously prompting the environmental profile of the method^26^. Analyte retention, peak shape, resolution, and ionization efficiency are highly affected by the choice of mobile phase, particularly in multi-analyte UPLC–MS/MS assays involving compounds with diverse polarity and ionization characteristics. Therefore, solvent selection must accomplish a balance between elution strength and interaction with the stationary phase, while maintaining compatibility with electrospray ionization^27^. In parallel, greenness considerations are integrated at this stage by evaluating solvent’s toxicity, environmental impact, and safe handling profiles^28^. Analytical performance is achieved without compromising environmental and safety aspects by embedding greenness within mobile phase selection, in this manner strengthening the overall robustness and sustainability of the proposed optimization strategy.

### Step 5: method validation

The next stage of the proposed optimization strategy focuses on method validation to determine that all selected experimental conditions provide reliable and dependable analytical performance. This step confirms that prior decisions associated to extraction, chromatographic conditions, and mass spectrometric parameters translate into a robust and reliable method. Validation should cover key performance characteristics, containing selectivity, linearity, accuracy, precision, recovery, matrix effect, and stability. Linearity should be displayed with correlation coefficients typically exceeding 0.99, while calculated concentrations remain within acceptable variation limits. Multiple samples of blank biological matrix were used to assess selectivity to ensure the absence of interference, particularly at the lower limit of quantification. Extraction recovery should be reliable and reproducible, even if not quantitative, as variability in recovery directly impacts method reliability. Different concentration levels over multiple runs are measured to confirm high level of accuracy, and precision under routine conditions. In addition, estimation of ion suppression or enhancement caused by co-eluting matrix components expressed as matrix effect evaluation is crucial to be within acceptable limits to ensure signal stability. Finally, stability studies under various conditions must confirm that analytes remain unchanged throughout sample handling, processing, and storage.

### Step 6: greenness evaluation and sustainability consideration

As the final step of the proposed stepwise model, a multidimensional sustainability and performance evaluation was implemented to move beyond reliance on a single assessment perspective^29,30^. The Red Analytical Performance Index (RAPI) was employed to examine analytical performance through criteria related to precision, trueness, recovery, sensitivity, working range, linearity, robustness, and selectivity^31^. whereas the Blue Applicability Grade Index (BAGI) addressed the practical feasibility and operational applicability of the analytical workflow^32^. Environmental performance was assessed using complementary green metrics, including the Modified Green Analytical Procedure Index (MoGAPI), the Analytical GREEnness metric (AGREE), and the Analytical Green Star Assessment (AGSA), which collectively examine different aspects of reagent hazards, waste generation, energy demand, sample preparation, procedural safety, and overall environmental burden^33–35^. Finally, the RGB12 model was applied to integrate the red, green, and blue dimensions within a unified White Analytical Chemistry framework, thereby providing a broader view of analytical quality, environmental responsibility, and practical efficiency^36^. The combined implementation of RAPI, BAGI, MoGAPI, AGREE, AGSA, and RGB12 enables a more comprehensive evaluation of the developed method and supports balanced decision-making rather than optimization based solely on analytical performance or greenness.

## Experimental

All experimental procedures and analytical methods were performed in accordance with the relevant institutional guidelines and regulations. Human plasma used in this study was commercially obtained and utilized only for spiking purposes; therefore, no human participants or volunteer recruitment were involved, with an approved ethics form provided from MSA university ( Ref. No.: A7/Ec7/2022F). The MSA Ethics Committee waived the requirement for informed consent from all participants or their legal guardians, including consent for the use of plasma samples.

### Reagents and chemicals

RIF was provided by Al-Andalous Co. (6 October, Egypt), CIP was supplied by Memphis Co. for Pharm while FLU was supplied by Pfizer, Egypt. Authentic standard drugs’ purity was evaluated using drugs’ official British Pharmacopoeia (BP) methods (British Pharmacopoeia, 2013). The solvents of HPLC grade were obtained; methanol was obtained from Lab Scan Limited Dublin Ireland. Blank human plasma used for spiking was purchased from Holding Company for Biological Products & Vaccines (VACSERA). The plasma provided by VACSERA is collected, processed, and distributed in accordance with national regulations. Importantly, all samples supplied to researchers are fully anonymized and de-identified before distribution, with no associated personal, demographic, or clinical data accessible to the authors. Therefore, no individual could be identified, and the study did not involve any direct interaction with human subjects.

### Instrument

Chromatographic analysis was performed with a binary solvent management pump and UPLC MS/MS “Waters” 3100 “USA”. Analysis was accomplished using a binary solvent manager (Acquity ultra performance LC) and TQ triple quadrupole mass spectrometer (Acuity Ultra performance LC) with both positive and negative electrospray ionization (ESI). Data acquisition and instrument control were performed using MassLynx software (version 4.1; Waters Corp., Milford, MA, USA).

### Mass spectrometric and liquid chromatographic used conditions

Chromatographic separation for all three drugs was carried out using a reverse-phase column; Agilent Poroshell^®^ C18 with dimensions (4.6 × 50 mm, 2.7 μm particle size). Mobile phase transfer was handled by a binary pump operating under isocratic conditions, using a methanol/water mixture (95:5, v/v). A flow rate of 0.5 mL/min was used during analysis. Detection utilizing mas spectrometric was performed on a TQ instrument, which proves MRM acquisition and corresponds with electrospray ionization modes both positive and negative. Before use, the mobile phase was degassed through ultrasonication. The mass spectrometer was calibrated using the auto-tune function within MassLynx V4 software. Quantitative analysis was accompanied in MRM mode, with the following ion transitions monitored: m/z 786.55 → 754.44 for RIF, m/z 332.27 → 230.93 for CIP, m/z 307.23 → 238.17 for FLU, and m/z 205.15 → 160.86 for the internal standard. Mass spectrometric parameters evaluated are presented in Table 1.

**Table 1:** LC/MS–MS parameters selected for the quantification of Rifaximin, Ciprofloxacin and Fluconazole using Ibuprofen as internal standard.

Analyte	Parent (m/z)	Danghter (m/z)	Dwell (Sec)	Cone (V)	Collision energy (eV)	Ion mode
Rifaximin	786.55	754.44	0.058	35	20	+ve
Ciprofloxacin	332.27	230.93	0.058	30	35	+ve
Fluconazole	307.23	238.17	0.058	20	15	+ve
Ibuprofen (IS)	205.15	160.86	0.058	20	7	−ve

### Working and standard solutions preparation

Individual stock solutions of RIF, CIP, and FLU were separately prepared in methanol at concentrations of 10, 100, and 100 µg/mL, respectively. From these stock solutions, individual working standard solutions were prepared by appropriate dilution with methanol at concentrations of 1 µg/mL for RIF, 25 µg/mL for CIP, and 40 µg/mL for FLU.

### Construction of calibration curve

To prepare calibration samples, Working solutions with volume of 50 uL were mixed with 450 µL of human plasma were used to prepare the calibration samples. This resulted in final plasma concentrations of 5–100 ng/mL for RIF, 200–2200 ng/mL for CIP, and 400–3200 ng/mL for FLU. Regarding the Quality Control samples, prepared at three levels in plasma: low (LQC), medium (MQC), and high (HQC). The corresponding QC concentrations were set at 20, 50, and 80 ng/mL for RIF; 600, 1000, and 1800 ng/mL for CIP; and 800, 1400, and 2400 ng/mL for FLU.

### Preparation of samples

Preparation is accomplished via mixing of IS solution with 300 µL of human plasma for 1 min. As for extraction, 4 mL of dichloromethane is mixed with the previously prepared solution for 12 min followed by centrifugation for 10 min at 5000 rpm. A volume of 3.2 mL was then separated for drying under nitrogen atmosphere at 40 °C. 400 µL of mobile phase were used for reconstitution. determination was done using UPLC-MS/MS system by injection of 5 µL of reconstituted sample.

## Results and discussion

### Step 1: physicochemical profiling

Preliminary estimation of dissolution characteristics, molecular weight, hydrophilicity, and plasma protein affinity constructs a coherent framework for predicting chromatographic and detection performance in spiked plasma. RIF was expected to show high hydrophobic retention caused by high molecular weight, logP of 4.94, and pKa of 3.66–11.87 for strongest acidic and strongest basic respectively^37^. While CIP, presenting average polarity and anticipated to demonstrate pH-dependent distribution behavior due to zwitterionic character due to logP < 1 and pka values of 6 and 8.8^38^. FLU was assumed to undergo effective isolation yet potentially demonstrate a divergent ionization response relative to the remaining compounds due to it hydrophilicity nature and slightly protein-associated with pKa value of 2.03 and logP of 0.5^39^. All three target analytes resulted in optimal signals under positive-mode electrospray ionization, aligned with their proton-accepting capabilities. The observed lower quantification threshold of 5 ng/mL for RIF versus 200 ng/mL for CIP and 400 ng/mL for FLU resulted from alterations in ionization efficiency and mass spectrometric sensitivity during polarity switching rather than extraction. This confirms that early physicochemical evaluation supported the prediction of sensitivity variability and managed instrument method optimization.

### Step 2: Extraction strategy selection

LLE was specifically suitable considering the physicochemical diversity of the analytes, where RIF exhibits moderately high lipophilicity and poor aqueous solubility, whereas CIP and FLU display moderate polarity. It was nominated as the sample preparation technique in order to accomplish efficient matrix cleanup and consistent recovery for simultaneous determination of the studied analytes in plasma. Dichloromethane is a non-polar solvent with robust extraction ability for moderately lipophilic compounds. This permitted simultaneous extraction of all three analytes regardless of their polarity differences. The combination of adequate recovery and acceptable matrix factor confirms that dichloromethane extraction provided sufficient removal of endogenous components.

### Step 3: chromatographic optimization

The highly organic nature of analytes prefers the use of a C18 reversed-phase column (4.6 × 50 mm, 2.7 μm) during chromatographic separation, accompanied by using isocratic elution with methanol/water mixture (95:5, v/v) at a flow rate of 0.5 mL/min. These conditions enabled rapid elution and narrow peak widths while maintaining sufficient separation among analytes. RIF, CIP, and FLU were monitored in positive electrospray ionization mode due to their molecular structures, which contain basic functional groups that tend to protonation. RIF possesses multiple heterocyclic nitrogen atoms that readily accept protons, while CIP contains a piperazinyl moiety with strong basic character. FLU also contains triazole rings that display proton affinity. These structural characters raise the formation of stable [M + H]⁺ ions, thereby improving ionization efficiency and sensitivity in positive mode. Ibuprofen was monitored in negative mode due to the existence of a carboxylic acid functional group, which readily undergoes deprotonation to form a stable [M − H]⁻ ion. It was employed as a non-isotopic internal standard based on its experimentally satisfactory extraction recovery, stable MS response, distinct MRM transition, and absence of relevant interference under the optimized analytical conditions. It demonstrated satisfactory performance during the adopted liquid–liquid extraction procedure, with an extraction recovery of 103.65%, while no relevant interference was observed at its detection region. This involves polarity switching within the 3-minute acquisition window. A dwell time of 0.058 s per transition was applied to maintain sufficient data points across each chromatographic peak. Despite polarity switching, signal stability remained adequate.

### Step 4: mobile phase selection

Regarding mobile phase selection, 2 trials have been done to select a suitable combination form: methanol/water mixture (95:5, v/v) or methanol/formic acid (95:5, v/v). A superior chromatographic and mass spectrometric performance was achieved by using methanol/water mixture (95:5, v/v) compared with methanol/formic acid (95:5, v/v) for method development. Methanol/water (95:5, v/v) was selected based on its compatibility with the physicochemical properties of the studied analytes. The high methanol content provides strong elution strength, which is essential for efficiently eluting the highly lipophilic analytes and preventing peak broadening. The inclusion of water ensured well-defined and resolved peaks through adequate control of analyte retention and allowed proper interaction with the stationary phase. In contrast, replacing water with a high proportion of formic acid elevated ionic strength, which adversely affected electrospray ionization by promoting ion suppression and reducing signal stability. This interpretation is supported by previous studies demonstrating that ESI efficiency is strongly dependent on mobile-phase composition, pH, organic-phase content, and analyte-specific physicochemical properties, with mobile-phase additives capable of producing substantial and compound-dependent changes in MS response^40^. Moreover, methanol content can influence electrospray droplet properties, including surface tension and evaporation behavior, which directly affect signal intensity^26^. Overall, methanol/water (95:5, v/v) achieved a better balance between chromatographic separation and ionization efficiency, making it more suitable for rapid and reliable multi-analyte UPLC–MS/MS analysis.

#### Comparative greenness assessment and mobile phase selection based on spider diagram analysis

Mobile phase selection was guided by a comparative greenness assessment using spider diagram analysis of two solvent systems: methanol/formic acid and methanol/water^41^. The methanol/water system exhibited a significantly higher overall greenness score (4.164) than the methanol/formic acid system (0.26), indicating a markedly improved environmental and safety profile (Fig. 2a). This enhancement was driven by higher scores in stability, fire safety, odor, and health impact, reflecting the non-hazardous, non-flammable, and odor-neutral nature of water. While both systems showed comparable contributions in general properties. The incorporation of formic acid significantly degraded the overall greenness due to its corrosivity and associated handling risks. In contrast, the methanol/water system maintained a more balanced and consistently high scoring profile across all evaluated criteria, resulting in a larger and more uniform spider diagram area. Therefore, the selection of a methanol/water mixture (95:5, v/v) was justified not only by its enhanced sustainability profile but also by its ability to deliver reliable and efficient chromatographic performance.

#### Green solvent selection tool (GSST)

The suitability of the selected methanol/water mixture (95:5, v/v) mobile phase was further supported by Hansen solubility parameter analysis^28^. As illustrated in the Hansen space, the position of the solute (dD = 15.1, dP = 14.2, dH = 32.3) indicates a system with significant hydrogen bonding and moderate polarity requirements (Fig. 2b). The synergistic behavior of methanol and water explains the effective dissolution, stable chromatographic performance, and improved peak characteristics observed experimentally. Moreover, the yellow and green color distribution of methanol and water in the Hansen map reflects their favorable sustainability profiles, further supporting their selection as a functional and environmentally preferable mobile phase.

### Step 5: method validation

#### Method development and optimization

**Fig. 3:**
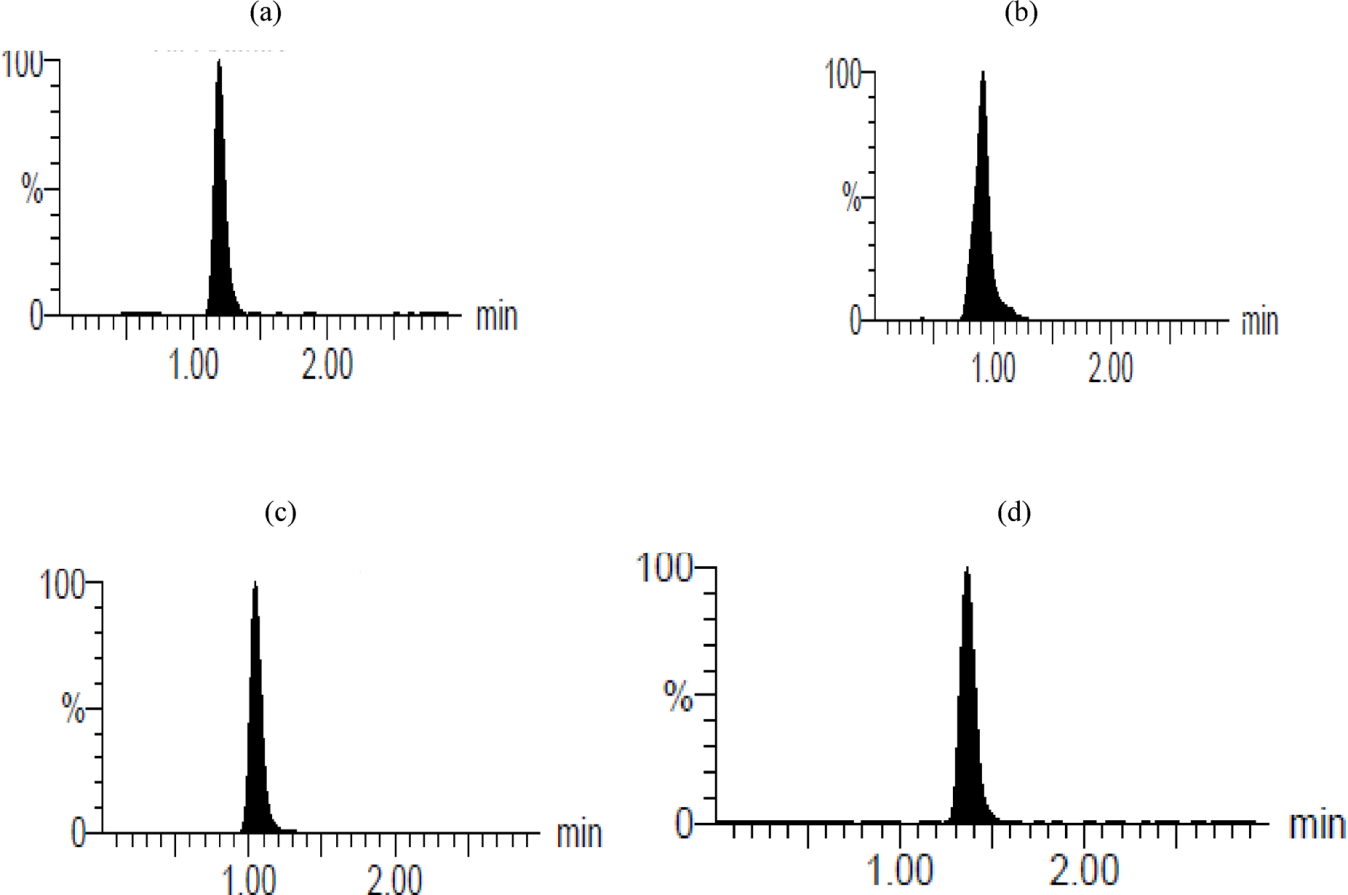
Mass chromatograms of plasma spiked with (**a**) RIF (**b**) CIP, (**c**) FLU, and (**d**) IS using isocratic elution of methanol and water (95:5, v/v) at flow rate 0.5 mL/min.

Bioanalysis quantification of measured drugs at low concentration level in plasma with good acceptable recovery is effective for bioequivalence, therapeutic drug monitoring, bioavailability. The optimum separation of analytes with clear resolved peaks without tailing (Fig. 3) was accomplished by using a mobile phase of methanol/water mixture (95:5, v/v) that eluted isocratically, at flow rate of 0.5 ml/min and Agilent Poroshell^®^ C18 with dimensions (4.6 × 50 mm, 2.7 μm particle size) column. The positive ion mode was chosen for MRM analysis of RIF, CIP and FLU while for IS negative ion mode was selected for analyte detection by mass spectrometry. The protonated precursor parent ions [M + H]^+^ were at m/z 786.55 for RIF, 332.27for CIP, 307.23 for FLU and 205.15for IS. The subsequent product ions were selected at 754, 230, 238 and 160 for RIF, CIP, FLU and IS, respectively (Figure S2). For the extraction of drugs from plasma matrix, a LLE procedure using dichloromethane as solvent showing good reproducible recovery % (103.65%) with minimum matrix effect for RIF, CIP, FLU and IS.

#### Selectivity

Six blank plasma samples were chromatographically inspected and compared to plasma samples spiked with the three analytes and the internal standard. The results approved that no endogenous interferences were detected at the retention times of the compounds of interest. Additionally, background noise levels were evaluated and determined to be below 20% of the lower limit of quantification for RIF, CIP, and FLU, and below 5% of the internal standard response, as illustrated in Figure S2.

#### Linearity and range

Linearity was accomplished through structured calibration curves in range of (5-100 ng/mL) for RIF, (200–2200 ng/mL) for CIP, and (400–3200 ng/mL) for FLU. Estimation of interference from human plasma was detected using blank plasma (without IS) and the zero plasma (with IS), indicating no interference with the three studied drugs Table S1. An adequate deviation from the nominal concentration (within ± 15%) was obtained by HQC, MQC, and LQC.

#### Accuracy and precision

Six replicates of four levels of concentration (LLOQ, LQC, MQC, and HQC) of RIF, CIP, and FLU were utilized for the determination of the intra-day and inter-day accuracy and precision. The intra-day accuracy differed between 103.56 and 102.36% with RSD of 3.48–7.64% for RIF, 99.67-103.91% with RSD of 4.11–3.74% for CIP and 99.27-103.98% with RSD of 4.29–6.67% for FLU. While the inter-day accuracy was expressed in range of 107.45-105.96% with RSD of 7.23–4.97% for RIF,101.19-103.33% with RSD of 9.41–3.67% for CIP and 103.87-101.33% with RSD of 8.34–3.87% for FLU as shown in Table 2.

**Table 2:** Intra- and inter‐day accuracy and precision for the determination of Rifaximin, Ciprofloxacin and Fluconazole in spiked Human plasma.

Studied drugs	QC Level	Intra-day, *n* = 6		Inter-day, *n* = 6 × 3	
		Accuracy % ^a^	RSD % ^a^	Accuracy % ^a^	RSD % ^a^
Rifaximin	LLQC (5 ng/mL)	103.56	3.48	107.45	7.23
	LQC (20 ng/mL)	100.23	5.27	98.74	8.47
	MQC (50 ng/mL)	104.97	6.37	104.72	2.11
	HQC (80 ng/mL)	102.36	7.64	105.96	4.97
Ciprofloxacin	LLQC (200 ng/mL)	99.67	4.11	101.19	9.41
	LQC (600 ng/mL)	105.33	7.19	107.34	4.23
	MQC (1000 ng/mL)	100.97	5.34	102.37	5.41
	HQC (1800 ng/mL)	103.91	3.74	103.33	3.67
Fluconazole	LLQC (400 ng/mL)	99.27	4.29	103.87	8.34
	LQC (800 ng/mL)	102.36	7.15	109.34	7.36
	MQC (1400 ng/mL)	105.98	6.71	108.44	6.33
	HQC (2400 ng/mL)	103.98	6.67	101.33	3.87

^a^Average of six separate determinations.

#### Extraction recovery

Six replicates were used to evaluate extraction recovery by assessing the peak areas of all medications for the pre-extracted samples at three levels of LQC, MQC, and HQC, and comparing them to the peak areas of the medicines for the post-extracted plasma samples. The recovered materials demonstrate how effective the extraction process was. The average recovery for RIF was 104.99% with an RSD of 6.27%, for CIP it was 100.03% with an RSD of 5.64%, and for FLU it was 100.37% with an RSD of 8.42% as displayed in Table 3.

**Table 3:** Extraction recovery and matrix effect data for the determination of Rifaximin, Ciprofloxacin and Fluconazole in human plasma using ibuprofen as internal standard.

	QC level	Rifaximin		Ciprofloxacin		Fluconazole		Ibuprofen IS	
		Recovery% ^a^	RSD ^a^	Recovery% ^a^	RSD ^a^	Recovery% ^a^	RSD ^a^	Recovery% ^a^	RSD ^a^
Extraction recovery	LQC	107.64	5.26	96.84	4.65	101.86	7.23	103.65	5.36
	MQC	102.87	6.24	105.23	5.81	103.33	9.55		
	HQC	104.46	7.33	107.03	6.48	104.94	8.49		
Matrix effect	LQC	104.79	6.37	105.16	5.11	109.53	8.83	105.36	6.87
	MQC	99.58	5.97	110.03	7.25	108.67	6.34		

^a^Average of six separate determinations.

#### Matrix effect

Matrix effects were measured using six blank plasma batches by assessing the peak areas obtained from post-extracted plasma samples and compare them to those of plasma samples spiked with the reference drugs and internal standard at equivalent concentrations, across both low- and high-quality control levels. The outcomes displayed that any co-eluting matrix components did not interfere with the ionization of RIF, CIP, FLU, or the internal standard in the ion source. In addition, the records presented in Table 3 further support the efficiency of the sample preparation procedure in removing potential matrix-related interferences.

#### Stability studies

The stability of a drug in biological fluids is affected by several factors, including the drug’s chemical characteristics, storage conditions, and the composition of the matrix. In this study, drug stability in human plasma was assessed by calculating the percentage recovery (± RSD). The presented findings in Table 4, confirmed the stability of RIF, CIP, and FLU in plasma samples, as all recorded results remained within acceptable criteria.

**Table 4:** Stability study of Rifaximin, Ciprofloxacin and Fluconazole in human plasma by the proposed UPLC/MS/MS method.

Stability parameters	Rifaximin		Ciprofloxacin		Fluconazole	
	Plasma conc. (ng/ mL)	Recovery % ± RSD ^a^	Plasma conc. (ng/ mL)	Recovery % ± RSD ^a^	Plasma conc. (ng/ mL)	Recovery % ± RSD ^a^
Short term (6 h, room temperature)	20	98.11 ± 4.76	600	92.22 ± 2.34	800	89.22 ± 3.84
	80	90.36 ± 3.67	1800	90.34 ± 3.21	2400	95.32 ± 2.22
Post-preparative (24 h, room temperature)	20	91.44 ± 5.49	600	93.88 ± 4.54	800	93.19 ± 4.33
	80	93.33 ± 2.87	1800	90.37 ± 3.12	2400	92.49 ± 3.22
Freeze – thaw (three cycles)	20	91.61 ± 3.93	600	91.11 ± 3.08	800	88.33 ± 1.99
	80	92.55 ± 2.82	1800	91.55 ± 2.52	2400	91.03 ± 2.64
Long term (– 86 °C, 30 days)	20	88.37 ± 3.65	600	89.32 ± 5.36	800	88.74 ± 3.16
	80	89.51 ± 4.47	1800	93.77 ± 3.26	2400	92.65 ± 4.47

^a^Average of six separate determinations.

### Step 6: greenness evaluation and sustainability consideration

A comparative evaluation using six complementary assessment tools demonstrated that the proposed method achieved a balanced position relative to previously reported chromatographic approaches (Table 5). From the analytical performance perspective, the RAPI score of 70.0 indicated moderate performance compared with the reference methods, some of which achieved higher scores, reflecting differences in validation depth, sensitivity, precision, and selectivity^42–47^. Similarly, the BAGI score of 75.0 supported satisfactory practical applicability, although variations among the compared methods could be attributed to differences in sample throughput, automation, sample preparation, and operational requirements. Regarding environmental performance, the proposed method showed a favorable profile, achieving a MoGAPI score of 79 and an AGREE score of 0.77, which compared well with most of the investigated methods. In contrast, the AGSA score of 73.61 placed the method within a moderate sustainability range, reflecting the combined influence of solvent consumption, waste generation, energy demand, and procedural safety. Notably, the RGB12 assessment produced an overall whiteness score of 86.1, indicating effective integration of analytical performance, environmental considerations, and practical efficiency. Collectively, these findings show that although the proposed method did not attain the highest score for every individual metric, it maintained a consistent and competitive profile across the six assessment frameworks. This balanced performance is particularly relevant because optimization of one analytical dimension may compromise another, whereas the proposed method achieved an acceptable compromise among analytical reliability, environmental sustainability, and practical applicability.

**Table 5:**
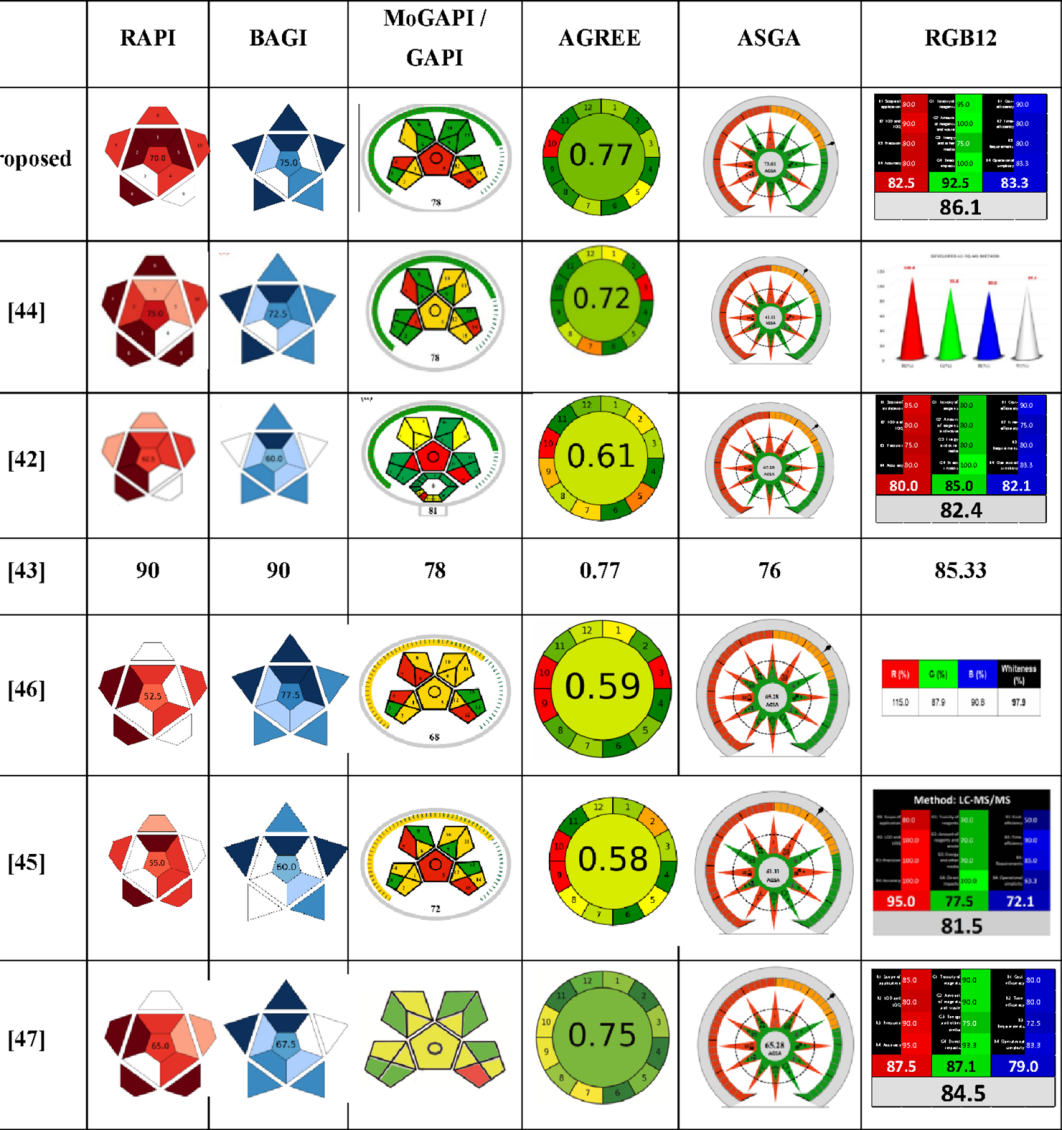
Comparative assessment of the proposed method and reported chromatographic methods using RAPI, BAGI, MoGAPI, AGREE, AGSA, and RGB12.

### Comparative evaluation of reported chromatographic studies within the stepwise optimization guidance model

The comparative analysis of previously reported chromatographic studies revealed substantial heterogeneity in the extent to which method development followed the proposed stepwise guidance model, as shown in Table S2. Physicochemical profiling was inconsistently addressed, with some studies considering isolated properties such as solubility or pH-dependent behavior, while systematic analyte profiling was absent from several reported studies. A similar fragmentation was observed for extraction strategy selection, where extraction conditions were frequently optimized without justification of the selection of extraction techniques. Chromatographic optimization represented the most consistently addressed stage and, in some studies, was supported by risk-based AQbD tools and experimental designs. However, systematic mobile-phase selection remained variable, ranging from comparison of different solvent systems to limited or absent screening. Method validation was consistently incorporated according to recognized guidelines, whereas greenness and sustainability assessments differed markedly in scope and were generally applied as endpoint evaluations using different combinations of assessment metrics. Collectively, this comparison indicates that the individual elements of the proposed model are established analytical practices, but their implementation across published studies remains fragmented and study dependent^42–47^. The contribution of the proposed framework therefore lies in organizing these elements into a sequential six-step decision pathway that begins with physicochemical profiling, uses this knowledge to inform extraction, chromatographic, and mobile-phase decisions, proceeds to guideline-based validation, and concludes with multidimensional evaluation of analytical performance, practical applicability, greenness, sustainability, and overall method balance. This structure also distinguishes the framework from conventional AQbD applications, which primarily strengthen risk assessment and experimental optimization within selected development stages.

## Conclusion

A structured six-step optimization guidance model was proposed for systematic development of rapid multi-analyte UPLC–MS/MS methods. The proposed framework integrates physicochemical profiling, extraction strategy selection, chromatographic optimization, mobile phase selection, method validation, and greenness evaluation within a unified analytical workflow. The model was designed to ensure that each stage of method development is scientifically rationalized and experimentally supported rather than relying only on final validation conclusions. Physicochemical profiling provides a preliminary understanding of analyte solubility, polarity, and ionization behavior, guiding subsequent optimization decisions. Extraction strategy selection and chromatographic optimization are aimed at improving recovery, minimizing matrix interference, and achieving rapid, efficient separation. Mobile phase selection is incorporated as an independent optimization step to balance chromatographic performance, ionization efficiency, and environmental sustainability through rational solvent selection. Method validation confirms the reliability and robustness of the developed assay according to regulatory expectations, while greenness evaluation assesses the environmental and practical impact of the analytical procedure. Overall, the proposed six-step model enhances transparency, reproducibility, and sustainability in UPLC–MS/MS bioanalytical method development and provides a transferable framework applicable to future multi-analyte analytical studies involving complex biological matrices.

## Supplementary Information

Below is the link to the electronic supplementary material.

Supplementary Material 1

## Data Availability

All data supporting the findings of this study are available within the paper and its Supplementary Information. The datasets used and/or analyzed during the current study are available from the corresponding author upon reasonable request.
