# Supplementary material for: A systematic stepwise optimization framework for rapid multi-analyte UPLC–MS/MS plasma analysis with integrated sustainability assessment
Source: Sci Rep. 2026 Jul 28;16:23496. doi: 10.1038/s41598-026-63587-9 (PMC13415790; doi:10.1038/s41598-026-63587-9)
Supplement: Supplementary file 1 — Supplementary Material 1 [file 41598_2026_63587_MOESM1_ESM.docx]

**Figure S1. Chemical structures of (a) Rifaximin, (b) Ciprofloxacin, (c) Fluconazole and (d) Ibuprofen (IS).**


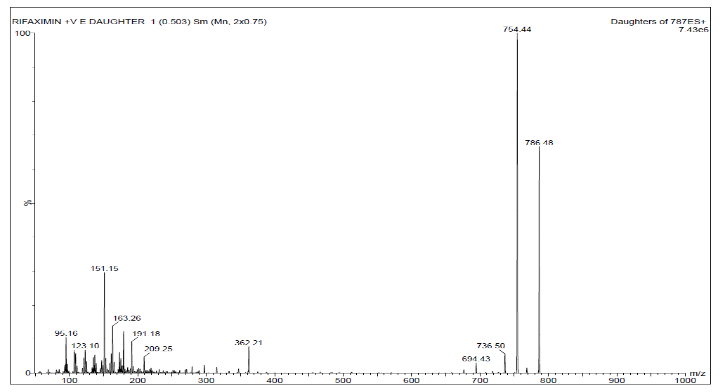

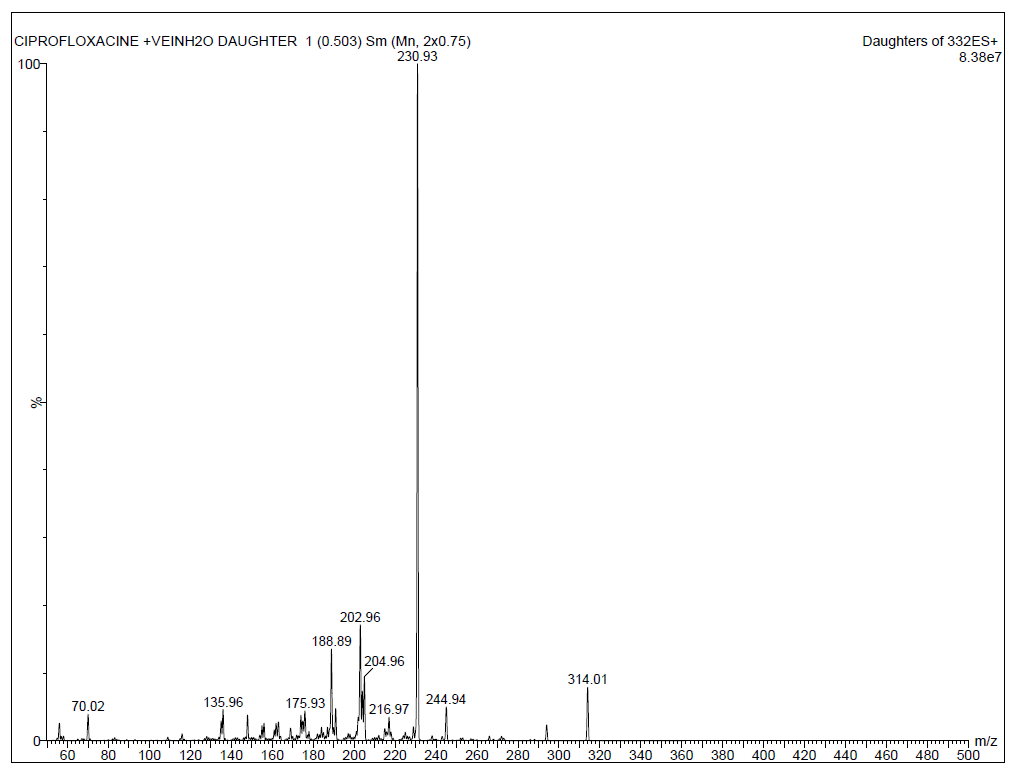


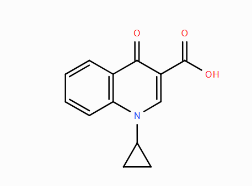


b

a


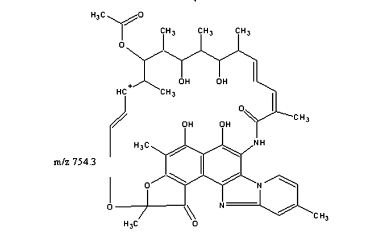


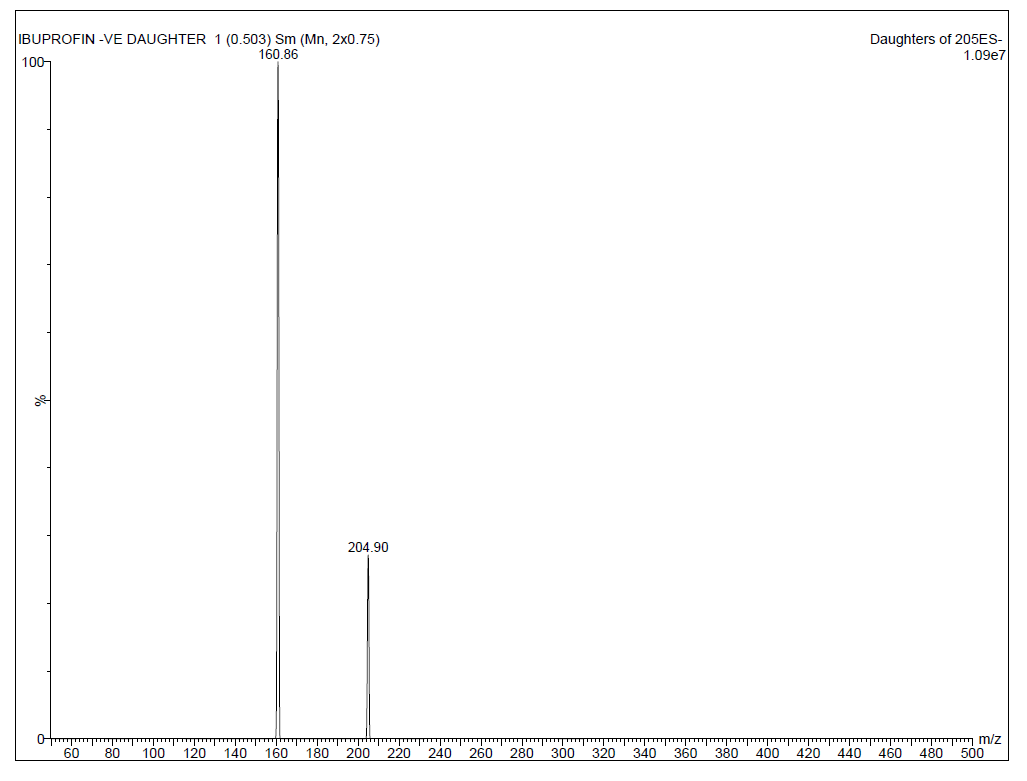

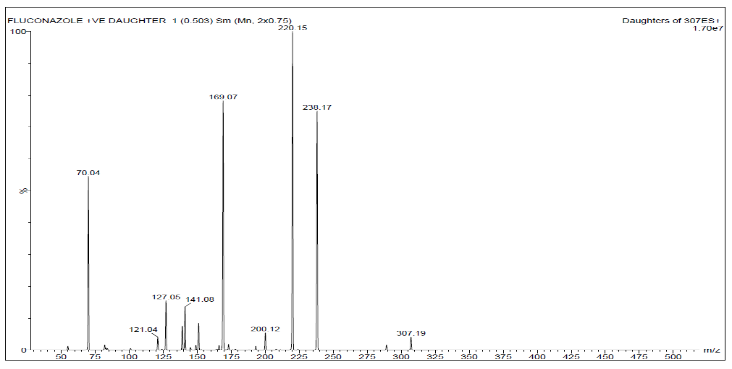

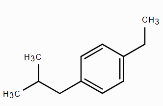

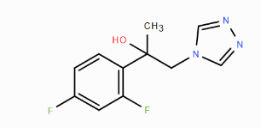


d

c

**Figure S2. Product ion spectra of [M + H] + of: The positive ion ESI-MS/MS spectra of (a) RIF 786.55< 754.44 m/z ; (b) CIP 323.27 < 230.93 m/z , (C) FLU 307.23< 238.17 m/z and (D) IS 205.15 < 160.86 m/z.**

**Table S1: Validation parameters of UPLC-MS/MS determination of RIF, CIP and FLU.**

| **Parameters** | **RIF** | **CIP** | **FLU** |
| --- | --- | --- | --- |
| **Correlation Coefficient** | **0.9973** | **0.9987** | **0.9985** |
| **Slope** | **0.0276** | **0.0058** | **0.1611** |
| **Intercept** | **-0.0509** | **2.3116** | **-52.216** |
| **SE on intercept** | **0.01319** | **0.10225** | **5.25786** |
| **LOD** | **1.577 (ng/ml)** | **58.179 (ng/ml)** | **107.703** |
| **LOQ** | **4.779 (ng/ml)** | **(176.301)** | **326.372** |
| **Regression equation** | **y = 0.0276x - 0.0509** | **y = 0.0058x + 2.3116** | **y = 0.1611x - 52.216** |

**Table S2. Comparative evaluation of reported chromatographic studies within the Stepwise Optimization Guidance Model**

| Steps | [44] | [42] | [43] | [46] | [45] | [47] |
| --- | --- | --- | --- | --- | --- | --- |
| Physicochemical Profiling | Considered Analyte solubility and pH-dependent behavior to guide sample preparation and MS ionization. | Considered Analyte solubility to explain solvent interactions. | No systematic profiling for any physicochemical profiling was reported. | No systematic profiling for any physicochemical profiling was reported. | Systematic profiling for physicochemical profiling was reported. | No systematic profiling for any physicochemical profiling was reported. |
| Extraction Strategy Selection | Extraction conditions were optimized without comparing different extraction techniques. | Justified microextraction over conventional SPE and LLE due to solvent use and workflow. | No systematic extraction-solvent screening was performed. | No systematic extraction-solvent screening was performed. | No systematic extraction-solvent screening was performed. | No systematic optimization of the extraction technique or selected solvents was reported |
| Chromatographic Optimization | Systematic optimization was performed using different columns depending on separation behavior. | Systematic optimization of column dimensions was reported. | Applied a strong risk-based AQbD workflow. | Systematic optimization was performed. | Systematic optimization was performed. | Applied a full AQbD workflow using Plackett–Burman design. |
| Mobile phase selection | Systematic optimization was performed using different volatile systems depending on peak shape. | No systematic optimization was reported. | No Systematic optimization for different systems was reported. | Systematic optimization was performed using different systems depending on peak shape. | Systematic optimization was performed using different systems | AQbD Systematic optimization for different systems, concentration and pH was reported. |
| Method Validation | Validation followed ICH guidelines. | Validation parameters have been assessed. | Validation followed ICH guidelines. | Validation followed ICH guidelines. | Validation followed ICH guidelines. | Validation followed FDA guidelines. |
| Greenness Evaluation and Sustainability Consideration | Applied AGREE, MoGAPI, BAGI, RAPI, CaFRI, and RGB12. | Applied ComplexMoGAPI, BAGI, VIGI, and RAPI. | Applied AGREE, Mo-GAPI, AGSA, RGB12, RAPI, and BAGI. | Applied AGREE, HEXAGON, and WAC or RGB12. | Applied RGB12. | Applied NEMI, modified NEMI, AMVI, Analytical Eco-Scale, AMGS, HPLC-EAT, GAPI, and AGREE. |
